# Unravelling the Anti-Inflammatory and Antioxidant Potential of the Marine Sponge *Cliona celata* from the Portuguese Coastline

**DOI:** 10.3390/md19110632

**Published:** 2021-11-12

**Authors:** Joana Alves, Helena Gaspar, Joana Silva, Celso Alves, Alice Martins, Fernando Teodoro, Patrícia Susano, Susete Pinteus, Rui Pedrosa

**Affiliations:** 1 MARE—Marine and Environmental Sciences Centre, Politécnico de Leiria, 2520-630 Peniche, Portugal; joana.abr.alves@gmail.com (J.A.); joana.m.silva@ipleiria.pt (J.S.); celso.alves@ipleiria.pt (C.A.); fjtdgm@gmail.com (F.T.); patricia_susano94@hotmail.com (P.S.); susete.pinteus@ipleiria.pt (S.P.); 2 BioISI—Biosystems and Integrative Sciences Institute, Faculty of Science, University of Lisbon, 1749-016 Lisbon, Portugal; 3 MARE—Marine and Environmental Sciences Centre, Escola Superior de Turismo e Tecnologia do Mar, Politécnico de Leiria, 2520-614 Peniche, Portugal; alice.martins@ipleiria.pt

**Keywords:** marine natural products, chronic diseases, marine sponges, oxidative stress, inflammation, RAW 264.7.6

## Abstract

Inflammation is a double-edged sword, as it can have both protective effects and harmful consequences, which, combined with oxidative stress (OS), can lead to the development of deathly chronic inflammatory conditions. Over the years, research has evidenced the potential of marine sponges as a source of effective anti-inflammatory therapeutic agents. Within this framework, the purpose of this study was to evaluate the antioxidant and the anti-inflammatory potential of the marine sponge *Cliona celata*. For this purpose, their organic extracts (C1–C5) and fractions were evaluated concerning their radical scavenging activity through 2,2-diphenyl-1-picrylhydrazyl radical (DPPH), ferric reducing antioxidant power (FRAP), oxygen radical absorbance capacity (ORAC), and anti-inflammatory activity through a (lipopolysaccharides (LPS)-induced inflammation on RAW 264.7 cells) model. Compounds present in the two most active fractions (F5 and F13) of C4 were tentatively identified by gas chromatography coupled to mass spectrometry (GC-MS). Even though samples displayed low antioxidant activity, they presented a high anti-inflammatory capacity in the studied cellular inflammatory model when compared to the anti-inflammatory standard, dexamethasone. GC-MS analysis led to the identification of *n*-hexadecanoic acid, *cis*-9-hexadecenal, and 13-octadecenal in fraction F5, while two major compounds, octadecanoic acid and cholesterol, were identified in fraction F13. The developed studies demonstrated the high anti-inflammatory activity of the marine sponge *C. celata* extracts and fractions, highlighting its potential for further therapeutic applications.

## 1. Introduction

Inflammation is a driving factor in many chronic diseases (CD), such as cancer, stroke, chronic respiratory diseases, cardiovascular disorders, diabetes, autoimmune diseases, and age-related conditions [1]. The organism’ protective response upon tissue damage leads to the activation of neutrophils and macrophages, producing an oxidative burst at the inflammation site as a form of protection against injury, infection, and stress. It is a crucial process for survival, where the body eliminates noxious factors, constructs a memory of the damaging agent, and promotes tissue repair and wound healing. Typically, inflammation-inflicted stress triggers a fast and short-term immune response, restoring the organism’s homeostasis (acute inflammation). However, when this response is excessive and prolonged (chronic inflammation), it leads to cellular death and tissue destruction [2,3].

Macrophages play a major role in the host immune defense system during infection and disease development, being responsible for the release of inflammation mediators via a series of exquisitely orchestrated pathways that are spatiotemporally regulated [4]. The most common types of mediators are vasoactive amines, nitric oxide (NO), prostaglandins, leukotrienes, cytokines, and chemokines.

Cytokines are secreted by a large variety of cell types, such as lymphocytes, macrophages, dendritic cells, and endothelial, epithelial, and connective tissue cells, playing a crucial role in the mediation and regulation of immune and inflammatory reactions [5]. Ideally, there is a homeostatic control, created through a balance between inflammatory and anti-inflammatory cytokines [6]. The most studied cytokines are TNF-α, ΙL-1β, IL-6, IL-8, IL-10, and IL-12, among others.

Nitric oxide (NO) is a key signaling proinflammatory mediator produced by nitric oxide synthases (NOS), which plays a vital role in the pathogenesis of inflammation [7]. Under various inflammatory stimuli, the inducible isoform (iNOS), expressed primarily by macrophages, leads to a high production of NO [8]. This enzyme is highly expressed upon activation of NF-κB as a response to various stimuli, such as TNF-α, IL-6, IL-1β, and lipopolysaccharides (LPS). Although the downregulation of these molecules contributes to homeostasis, several other mechanisms inhibit inflammation in an interplay manner, such as anti-inflammatory cytokines (e.g., IL-10). Since persistent inflammatory conditions seem to be involved in the etiology of many CD, targeting inflammation has proven to be an effective therapeutic approach for many pathologies, and thus, allowing the development of a new generation of drugs suitable for the treatment of unhealthy conditions, such as cancer, autoimmune disorders, and infectious diseases [9,10].

Inflammatory conditions are known to be strongly correlated with oxidative stress (OS). This condition is induced by free radicals, such as reactive oxygen species (ROS) and reactive nitrogen species (RNS), when there is an imbalance between the production and removal of these molecules, due to an overproduction and/or reduced ability to neutralize them or repair the resulting damage [11,12]. Oxidative stress and chronic inflammation, both strongly linked to several CDs, are closely interrelated, bearing a mutual causality relation. The perpetual existence of inflammatory mediators along with ROS in the organism’s system contributes to maintaining an environment where inflammatory diseases arise, and tumor cells can proliferate [13]. As a result, targeting inflammation and oxidative stress has proven to be an effective therapeutic approach in many CDs. Hence, antioxidant and anti-inflammatory drugs may have a broader role than what was thought initially, with new possibilities arising for the treatment and therapy of CDs, by blocking the inflammatory processes and targeting specific mediators [9,10,14,15].

Even though the administration of analgesics and nonsteroidal anti-inflammatory drugs (NSAIDs) plays an important role in the suppression of the inflammatory response, its prolonged use can bear serious complications. Therefore, a large focus has been placed on the potential of biologically active compounds as new natural analgesic and anti-inflammatory compounds, as these possess minimal collateral effects and significant health benefits [16].

Nature has revealed to be a promising source of new bioactive products, with fewer side effects, safer use, and environmental friendliness. Sessile organisms, such as marine sponges, have been targeted as vigorous sources of unparalleled structurally active secondary metabolites (SM) with multiple health benefits, revealing a broad spectrum of biological activities with therapeutical relevance [17,18]. To date, a wide range of marine natural product classes with bioactive properties have been isolated from sponges, including alcohols, alkaloids, amino acid derivatives, aromatic compounds, fatty acids, lactones, peptides, polyacetylenes, polyketides, quinones, quinolones, sphingolipids, sterols, terpenes, and terpenoids. Several marine sponge compounds are known to possess a remarkable anti-inflammatory capacity, with their specific mechanism of action being disclosed, in the majority of cases [19].

The marine sponge *Cliona celata* (Grant 1826) is commonly found throughout the Portuguese coast; however, the biotechnological potential of this species is yet to be thoroughly studied, as only few papers have reported the biological potential of *C. celata*, namely its anti-inflammatory and autophagy-modulating activity [20,21]. The goal of this work was to assess the in vitro antioxidant and anti-inflammatory potential of crude extracts and fractions from *C. celata*, to understand possible mechanisms of action, and determine the chemical profile by GC-MS analysis of the most promising samples.

## 2. Results

### 2.1. Evaluation of Biological Activities of Cliona celata Crude Extracts

Aiming a bioguided assay, the biological activities of *C. celata* crude extracts (antioxidant, cytotoxic, and anti-inflammatory activity) were primarily evaluated to select the most promising extract for fractionation.

#### 2.1.1. Antioxidant Activity

The antioxidant capacity of *C. celata* crude extracts was assessed through different assays: the free radical 2,2-diphenyl-1-picrylhydrazil (DDPH), the ability to reduce ferric iron (FRAP), and the oxygen radical absorbance capacity (ORAC). BHT (3,5-di-*tert*-4-butylhydroxytoluene) was used as standard. The results are displayed on Table 1.

As shown in Table 1, crude extracts exhibited a high percentage of DPPH values, displaying no significant differences when compared to the vehicle, and therefore, low radical scavenging ability, while BHT, the antioxidant standard, presented reduced percentage of DPPH values (39.09 ± 2.97%). On the FRAP assay, C4 displayed the highest capacity among extracts (77.63 ± 4.10 μM FeSO_4_ eq·g^−1^); however, the obtained values were approximately 36 times lower than the ones presented by BHT (2821.50 ± 63.04 μM FeSO_4_ eq·g^−1^). Contrarily to the previous methods, in the ORAC assay, except for C2, all crude extracts presented higher antioxidant capacity than BHT (136.38 ± 9.09 µmol T eq·g^−1^), with the highest capacity being displayed by C3 (248.91 ± 6.74 µmol T eq·g^−1^), followed by C4 (239.37 ± 7.50 µmol T eq·g^−1^). Overall, crude extracts presented a low antioxidant capacity (Table 1).

#### 2.1.2. Cytotoxic Activity in RAW 264.7 Macrophages

**Figure 1:**
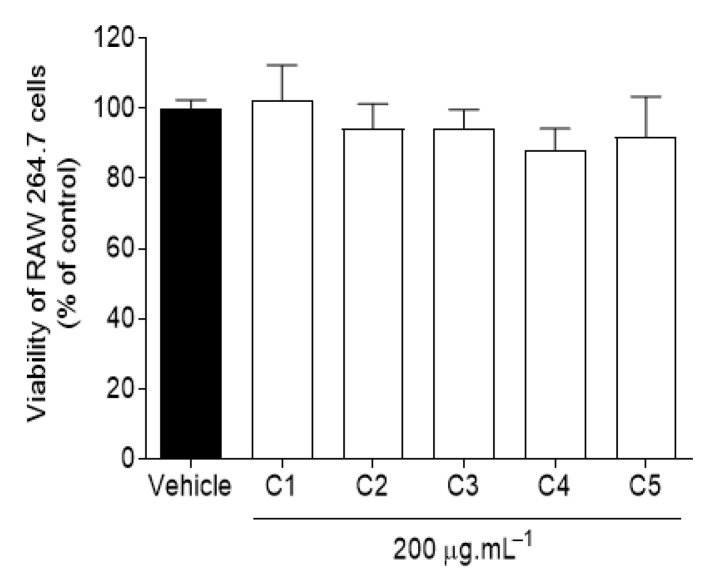
Cytotoxicity of *Cliona celata* crude extracts on RAW 264.7 macrophages. Cell viability was evaluated after 24 h of exposure to extracts (200 µg·mL^−1^) and the results are expressed as a percentage of the control. Bars correspond to mean ± SEM of at least three independent experiments carried out in triplicate. No significant differences (one-way ANOVA, Dunnett’s test; *p* < 0.05) were found when comparing the extracts to the vehicle.

The cytotoxic activity of *C. celata* crude extracts was evaluated on RAW 264.7 macrophages, beingthe obtained results depicted in Figure 1.

Data gathered from Figure 1 sustain that the viability of RAW 264.7 macrophages was not affected by *C. celata* crude extracts at a concentration of 200 μg·mL^−1^, thus revealing no cytotoxicity.

#### 2.1.3. Quantification of Nitric Oxide (NO) in RAW 267.4 Cells

**Figure 2:**
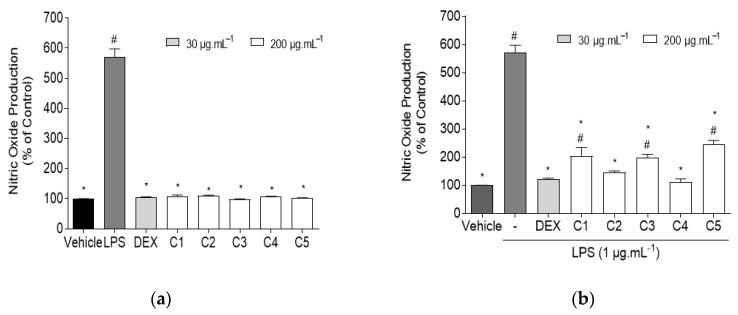
Evaluation of the inflammatory and anti-inflammatory potential of *Cliona celata* crude extracts (C1–C5) and DEX (dexamethasone) on RAW 264.7 macrophages. (**a**) NO production by RAW 264.7 macrophages when exposed to *C. celata* crude extracts (24 h) at 200 μg·mL^−1^; (**b**) NO production by LPS-induced RAW 264.7 macrophages in the presence of *C. celata* crude extracts (24 h) at 200 μg·mL^−1^. Bars correspond to mean ± SEM of at least three independent experiments carried out in triplicate. Symbols represent significant differences (one-way ANOVA, Dunnett’s test; *p* < 0.05) when compared to: ^#^ vehicle and * LPS.

RAW 264.7 macrophages were used as a model to assess the effect of *C. celata* crude extracts on the NO production in the presence and absence of inflammation induced with lipopolysaccharides (LPS). This approach allowed us to understand if the extracts were capable of inducing spontaneous inflammation on RAW 264.7 macrophages, or if they could reduce NO levels when exposed to an inflammatory condition. Dexamethasone (DEX) was used as an anti-inflammatory standard. The obtained results are shown in Figure 2.

Data gathered in Figure 2a suggest that *C. celata* crude extracts did not promote the production of NO in RAW 264.7 macrophages. No significant differences were encountered when comparing any of the extracts with the vehicle, thus confirming the existence of basal NO levels in cells, and the lack of inflammatory capacity of samples.

**Figure 3:**
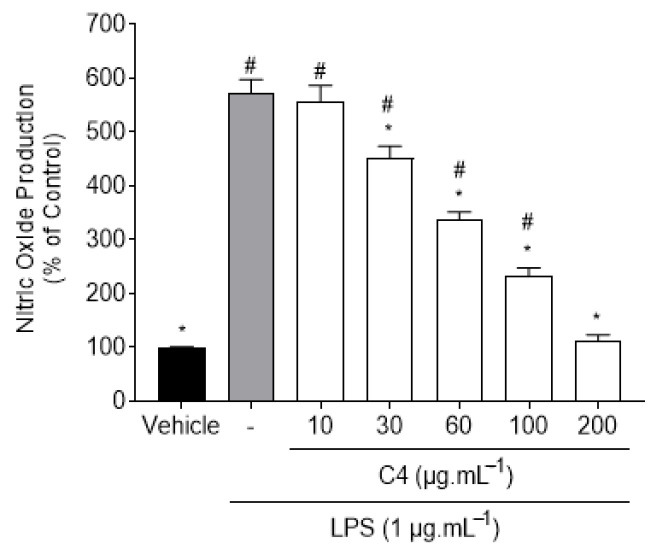
Evaluation of the concentration-dependent effect of C4 (24 h) in the NO production by LPS-induced RAW 264.7 macrophages, at concentrations ranging from 0 to 200 μg·mL^−1^. Bars correspond to mean ± SEM of at least three independent experiments carried out in triplicate. Symbols represent significant differences (one-way ANOVA, Dunnett’s test; *p* < 0.05) when compared to: ^#^ vehicle and * LPS.

LPS-exposed RAW 264.7 macrophages exhibited high levels of NO (570.50 ± 26.97%), characteristic of an inflammatory condition; however, upon treatment with the crude extracts, NO levels were significantly reduced when compared with the LPS group, as seen in Figure 2b. Extract C4 exhibited the highest anti-inflammatory potential (110.90 ± 12.08%), followed by C2 (145.40 ± 5.53%), both of which presented basal NO levels, as no significant differences were found when comparing C4 and C2 with the control group. DEX (30 μg·mL^−1^) also reduced NO production (123.20 ± 2.32%) to basal levels. Based on these findings, a concentration-dependent analysis was performed to assess the anti-inflammatory potential of the most promising extract (C4), and the results are displayed in Figure 3.

Extract C4 displayed concentration-dependent effects verified by the production of NO on LPS-induced RAW 264.7 macrophages. The highest inhibitory effect was obtained at the maximum tested concentration of 200 μg·mL^−1^, this being the only concentration which did not show significant differences when compared to the control group. However, even at a concentration of 30 μg·mL^−1^, the anti-inflammatory potential of C4 (451.77 ± 21.37%) was still evident, displaying significant differences in relation to the LPS group.

### 2.2. Evaluation of Biological Activities of Cliona celata Fractions

Based on the previous results, extract C4 was selected for further fractionation through preparative column chromatography, due to its high anti-inflammatory capacity. Due to the lack of antioxidant activity exhibited by the crude extracts, fractions were not tested for their antioxidant potential.

#### 2.2.1. Cytotoxic Activity of C4 Fractions in RAW 264.7 Macrophages

From the 16 fractions obtained through the chromatographic separation of C4, only samples with a weight superior to 11 mg were tested for their cytotoxic effect on RAW 264.7 macrophages. The yields of *C. celata* fractions (F1–F14.2) and their cytotoxic capacity on RAW 264.7 macrophages at concentrations of 200, 160, and 100 μg·mL^−1^ are presented in Table 2.

As shown in Table 2, due to the low yields of F1-F4 and F10 (<1%), these fractions were not considered for the outlined in vitro bioassays. Data gathered in Table 2 suggest that RAW 264.7 macrophages’ viability was significantly affected by *C. celata* fractions. At the maximum concentration of 200 μg·mL^−1^, all fractions presented significant differences when compared with the control. However, at 160 μg·mL^−1^, fraction F5 (118.35 ± 2.83) did not induce a cytotoxic effect on RAW 264.7 macrophages. Similarly, at 100 μg·mL^−1^, no significant differences were encountered in relation to the control for F5 (101.00 ± 7.16) and F13 (82.61 ± 4.46).

#### 2.2.2. Effect of C4 Fractions (F5 and F13) on Nitric Oxide (NO) Levels in Normal and LPS-Induced RAW 267.4 Cells

**Figure 4:**
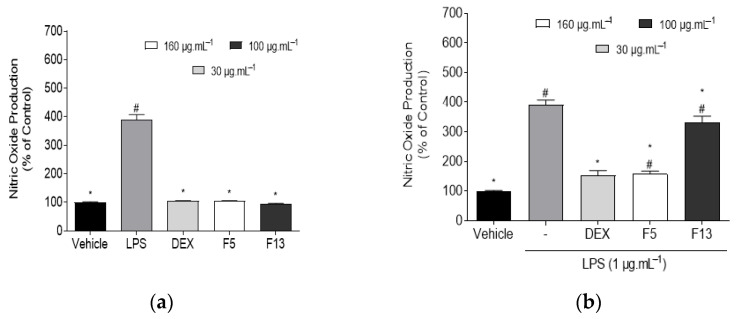
Evaluation of the inflammatory and anti-inflammatory potential of *Cliona celata* selected C4 fractions (F5 and F13) and DEX (dexamethasone) on RAW 264.7 macrophages. (**a**) NO production by RAW 264.7 macrophages when exposed to *C. celata* selected fractions; (**b**) NO production by LPS-induced RAW 264.7 macrophages in the presence of *C. celata* selected fractions (24 h). Bars correspond to mean ± SEM of at least three independent experiments carried out in triplicate. Symbols represent significant differences (one-way ANOVA, Dunnett’s test; *p* < 0.05) when compared to: ^#^ vehicle and * LPS.

The effect of F5 and F13 fractions, at non-cytotoxic concentrations, on NO production levels was evaluated on RAW 264.7 macrophages, in the presence and absence of LPS. The results are expressed on Figure 4.

Data gathered in Figure 4a suggest that fractions F5 and F13 were not capable of inducing the production of NO on untreated RAW 264.7 macrophages. No significant differences were found when comparing the vehicle with any of the fractions, thus confirming the existence of basal NO levels in RAW 264.7 macrophages, and the lack of inflammatory capacity of fractions.

Similarly to the behavior exhibited by *C. celata* crude extracts, treatment with the selected fractions led to a significant reduction of NO levels in relation to the LPS group, as seen in Figure 4b. Fraction F5 exhibited the highest anti-inflammatory capacity (157.32 ± 9.63%) at a concentration of 160 μg·mL^−1^, followed by F13 (332.45 ± 20.72%) at a concentration of 100 μg·mL^−1^. Fractions F5 and F13 and the standard drug DEX showed significant differences in relation to the LPS group; however, only DEX did not present significant differences when compared to the control group.

#### 2.2.3. Effect of C4 Fractions (F5 and F13) on the Levels of TNF-α, IL-6 and IL-10 on LPS-Induced Inflammation on RAW 264.7 Macrophages

**Figure 5:**
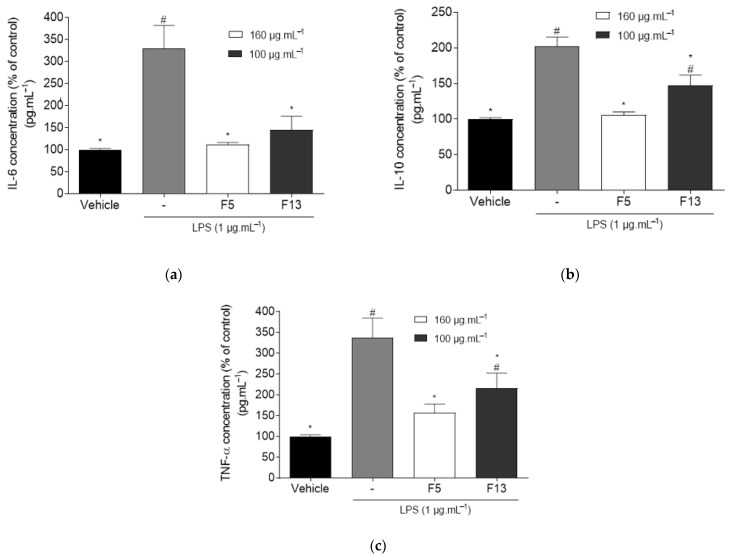
Effect of F5 (160 μg·mL^−1^) and F13 (100 μg·mL^−1^) on the levels of inflammatory mediators IL-6 (**a**), IL-10 (**b**), and TNF-α (**c**) on LPS-induced RAW 264.7 macrophages after 18 h of exposure. Bars correspond to mean ± SEM of at least three independent experiments carried out in triplicate. Symbols represent significant differences (one-way ANOVA, Dunnett’s test; *p* < 0.05) when compared to: ^#^ vehicle and * LPS.

The effect of fractions F5 and F13 on the levels of inflammatory mediators IL-6, IL-10, and TNF-α on LPS-induced inflammation on RAW 264.7 macrophages were assessed, and the results are shown in Figure 5.

Data gathered on Figure 5a show that LPS induction of inflammation leads to an increase in the levels of proinflammatory mediator IL-6 (329.18 ± 52.47%). Fractions F5 and F13 reduced the concentration levels of IL-6 (111.34 ± 4.95% and 145.01 ± 30.91%, respectively). No significant differences were encountered in relation to the vehicle with any of the fractions, thus confirming the existence of basal IL-6 levels on RAW 264.7 macrophages and the anti-inflammatory potential of fractions. As for Figure 5b, the expression of the anti-inflammatory mediator IL-10 is minimal in unstimulated tissues as seen for the basal levels in the vehicle group. However, LPS induction of inflammation leads to an increase in IL-10 levels (202.59 ± 12.75%) in the LPS group. Fraction F5 did not display significant differences when compared to the vehicle group (105.66 ± 4.32%); however, F13 presented higher levels of IL-10 (147.03 ± 14.85%), displaying significant differences with both the vehicle and LPS group. In Figure 5c, LPS induction of inflammation in the LPS group led to a marked increase in the concentration of pro-inflammatory mediator TNF-α (336.61 ± 47.48%). Treatment with F13 led to a reduction on the concentration of TNF-α (215.69 ± 36.62%), displaying significant differences with both the vehicle and LPS group. The highest anti-inflammatory potential was obtained by F5, which significantly reduced TNF-α concentration to basal levels (157.12 ± 20.60%) while not presenting significant differences with the vehicle group.

### 2.3. Chemical Characterization by GC-MS Analysis

The chemical profiles of the most bioactive fractions (F5 and F13) from *C. celata* were evaluated through GC-MS. Volatilized compounds were tentatively identified by matching their mass fragmentation patterns with those available in the GC-MS mass spectral databases (Wiley 229 and NIST-National Institute of Standards and Technology libraries). Compounds *n*-hexadecanoic acid, *cis*-9-hexadecenal, and 13-octadecenal were identified in fraction F5, while octadecanoic acid and cholesterol were detected in fraction F13 (Figure S1).

## 3. Discussion

Marine natural products isolated from sponges have shown potential to be applied in novel therapeutic approaches for CD [22] in great part due to their broad degree of chemical diversity and novel molecular structures with potential to counteract oxidative stress and inflammation [23]. In this study, the antioxidant activity of *C. celata* samples was evaluated by the means of three complementary assays, namely the scavenging capacity of the free radical DPPH, the ability to reduce ferric iron (FRAP), and the oxygen radical absorbance capacity (ORAC) displaying weak antioxidant capacity. Even though the antioxidant activity of marine sponge components is well-reported in the literature [24,25], there are currently no studies regarding the antioxidant potential of *Cliona celata*. A preliminary work conducted by Bary et al. (2016) on *Cliona viridis* also showed low scavenging potential by the DPPH method, which can suggest that the *Cliona* species do not produce metabolites with relevant antioxidant properties [26].

To evaluate the anti-inflammatory potential of *C. celata*, the first approach of this work aimed to understand if the extracts promoted inflammation by increasing the levels of NO. As none of the extracts induced NO production, the anti-inflammatory potential of *C. celata* crude extracts was evaluated in LPS-induced RAW 264.7 macrophages. At a concentration of 200 µg·mL^−1^, crude extracts displayed a marked effect on the reduction of NO levels, with extracts C4 and C2 exhibiting the higher anti-inflammatory capacity. A concentration-dependent assay was conducted for extract C4. Even though the anti-inflammatory potential of C4 ceased at a concentration of 10 µg·mL^−1^, there was still a significant effect on NO levels at 30 µg·mL^−1^. Under inflammatory stimuli, iNOS is highly expressed, leading to high levels of NO, which activate the inflammatory cytokines cascade. The expression of this enzyme is typically associated with the activation of NF-κB, which seems to be highly activated by other mediators, such as TNF-α and IL-6 [7]. Various marine sponge-derived components demonstrated unique and substantial anti-inflammatory potential by effectively reducing the levels of inflammatory mediators, such as NO, TNF-α, IL-6, and IL-1β, by downregulating inflammatory enzymes, namely iNOS and COX-2, and by modulating the signaling pathways that lead to the activation of NF-κB factor [27,28,29]. In agreement with the present results, a previous work conducted with *C. celata* ethyl acetate extracts also presented anti-inflammatory potential via iNOS regulation, even though the specimens were collected at very distinct locations [20].

The aforementioned results highlight the potential of *C. celata* crude extracts as promising sources of anti-inflammatory substances that can act in the mechanisms linked to NO production.

Due to its high anti-inflammatory activity, extract C4 was fractionated through a preparative column chromatography, producing 16 fractions, from which only 11 were tested for their cytotoxic effect in RAW 264.7 macrophages at concentrations of 200, 160, and 100 µg·mL^−1^. At a lower concentration, fractions F5 and F13 did not affect the viability of RAW 264.7 macrophages, thus being the only fractions tested for their anti-inflammatory activity. The enhanced cytotoxicity of fractions may be due to the higher concentration of cytotoxic compounds.

In a preliminary assessment, F5 and F13 fractions did not exhibit inflammatory activity, and thus, they were evaluated for their anti-inflammatory potential. Both fractions exhibited a high capacity of reducing NO levels in LPS-induced RAW 264.7 macrophages, showing higher anti-inflammatory potential than the crude extract C4, suggesting that the bioactive compounds were concentrated on these fractions.

Cytokines are powerful soluble immune mediators that can be used as target biomarkers to assess the role of inflammation in CD development [30]. TNF-α is a proinflammatory mediator responsible for mediating the expression of genes for growth factors, cytokines, transcription factors, and receptors, being involved in the pathophysiology of numerous diseases. Drug therapies that modulate TNF-α are widely used to treat inflammatory conditions [31,32]. IL-6 is a soluble mediator with a pleiotropic proinflammatory effect, which plays a role in the transition from acute to chronic inflammation. The dysregulated continual synthesis of IL-6 has shown to play a pathological effect on chronic inflammation and autoimmunity. IL-6 production is also enhanced in TNF-α and IL-1β activation of transcription factors. Strategies that target IL-6 and its signaling lead to effective prevention and treatment of chronic inflammatory diseases, such as rheumatoid arthritis [33,34]. IL-10 is a potent anti-inflammatory cytokine, which has been shown to inhibit the synthesis of many inflammatory proteins. Dysregulation of IL-10 is associated with enhanced immunopathology in response to infection as well as increased risk on the development of many autoimmune diseases. Recombinant human IL-10 proved to be effective in the control of inflammatory bowel disease (IBD) and psoriasis [35,36]. To get an insight on the mechanisms of action underlying the anti-inflammatory activities of *C. celata* fractions, the levels of TNF-α, IL-6 and IL-10 were quantified. The induction of the inflammatory condition with LPS treatment led to a marked increase on the concentration levels of pro-inflammatory mediator IL-6. Yet, treatment with F5 and F13 significantly decreased IL-6 concentration to basal levels, thus sustaining the anti-inflammatory capacity of these fractions. Similarly, in the TNF-α assay, LPS treatment led to high levels of this pro-inflammatory mediator. Treatment with F5 and F13 led to a reduction of the concentration levels of TNF-α, with the highest potential being exhibited by F5, which reduced the TNF-α concentration to basal levels. As a result, the anti-inflammatory potential of both fractions appears to be implicated in several regulatory pathways.

IL-10 is an anti-inflammatory mediator present in minimal concentrations in unstimulated tissues. As part of the organism’s innate regulatory mechanisms, the induction of inflammation leads to a natural increase in the production of anti-inflammatory mediators, which is supported by the increase in IL-10 levels in the LPS group. Cells exposed to LPS treated with F5 and F13 fractions displayed low levels of IL-10, especially in the case of F5, which did not display significant differences when compared to the control group. Since IL-10 is an enzyme that is activated later in the cytokine cascades [36], the anti-inflammatory effects of both fractions could be sufficient to inhibit the activation of the following mediators. These results strongly suggest that *C. celata* produces compounds with high anti-inflammatory potential, thus being extremely relevant to proceed with the bioactive compounds identification. Due to biomass constrictions, it was only possible to analyze the most promising fractions by GC-MS. Although this technique is directed to volatilized compounds, it can give valuable clues on the chemical composition of the extracts.

The anti-inflammatory activity of some marine sponges and their effect on TNF-α, IL-6, and IL-10 pathways has been previously reported [37,38]; however, it is important to highlight that this is the first report exhibiting the ability of *C. celata* to modulate anti-inflammatory mechanisms in the protein levels of TNF-α, IL-6, and IL-10.

The GC-MS analysis led to a tentative identification of some of the volatilized compounds present in fractions F5 and F13, as seen in the supporting information. Three compounds were matched in F5 fraction, namely *n*-hexadecanoic acid, *cis*-9-hexadecenal, and 13-octadecenal, and two in fraction F13, namely octadecanoic acid and cholesterol.

All of these compounds are quite common in nature, and although some have been pointed out as having anti-inflammatory potential, such as *cis*-9-hexadecenal due to being present in active extracts [39], no works were found specifically testing this compound’s anti-inflammatory potential. This evidence reinforces the relevance of further studies on *C. celata* to identify and test the isolated compounds. A future approach for further chemical investigation should be conducted by LC-HRMS/MS and NMR. Throughout the years, several compounds have been isolated from *C. celata*, namely clionamide A-D, celenamide A-D, and acetyl homoagmatine, but currently, no reported studies have investigated the antioxidant or anti-inflammatory activities of these compounds [21,40,41,42,43,44,45].

Through the present work, *C. celata* specimens collected from the Portuguese coast displayed compelling anti-inflammatory activity. Emphasis should be given to crude extract C4 and fractions F5 and F13, as these displayed the most promising anti-inflammatory activity. Data suggest that the specimen collected from the Arrábida site (C4) seems to reunite a unique set of chemical characteristics responsible for the enhanced anti-inflammatory capacity, with possible therapeutic application against chronic inflammatory diseases. Taking into account these new findings, it would be extremely relevant to retrieve more biomass from the Arrábida collection site for compounds isolation and structural characterization, as well as a deeper elucidation of their anti-inflammatory potential and mechanisms of action. It would also be of crucial significance to assess the effect of compounds in the mRNA and protein expression levels of more mediators, such as IL-1β, IFN-γ, PGE_2_, and COX-2, as well as the nuclear translocation and gene transcription activation of NF-κB. The present work presents the first assessment of anti-inflammatory activity of *C celata* collected on the Portuguese coast. Additionally, it is the first study assessing the mechanisms of action of *C. celata*, particularly on TNF-α, IL-6, and IL-10 pathways.

## 4. Materials and Methods

### 4.1. Chemicals and Reagents

Solvents of analytical and HPLC grades were purchased from VWR-BDH Chemicals (Fontenay-sous-Bois, France), Fisher Scientific (Loughborough, UK), and Honeywell Riedel-de-Haën (Illkirch, France), while ultrapure water was obtained from an Advantage A10 Milli-Q lab equipment (Merck, Darmstadt, Germany). Analytical grade chemicals and reagents from different suppliers were used to perform the in vitro bioassays, e.g., antioxidant capacity: Merck (Darmstadt, Germany), Sigma-Aldrich (Steinheim, Germany), and AlfaAesar (Karlsruhe, Germany); anti-inflammatory potential: Merck (Darmstadt, Germany), Sigma-Aldrich (St. Louis, MO, USA), Lonza (Basel, Switzerland), and Biowest (Riverside, CA, USA). Reagents and culture media for in vitro cellular assays were supplied by Merck (Darmstadt, Germany), Gibco (Grand Island, NY, USA), Invitrogen (Life Technologies, Warrington, UK), and Sigma (Seelze, Germany).

### 4.2. Marine Sponge Collection

**Figure 6:**
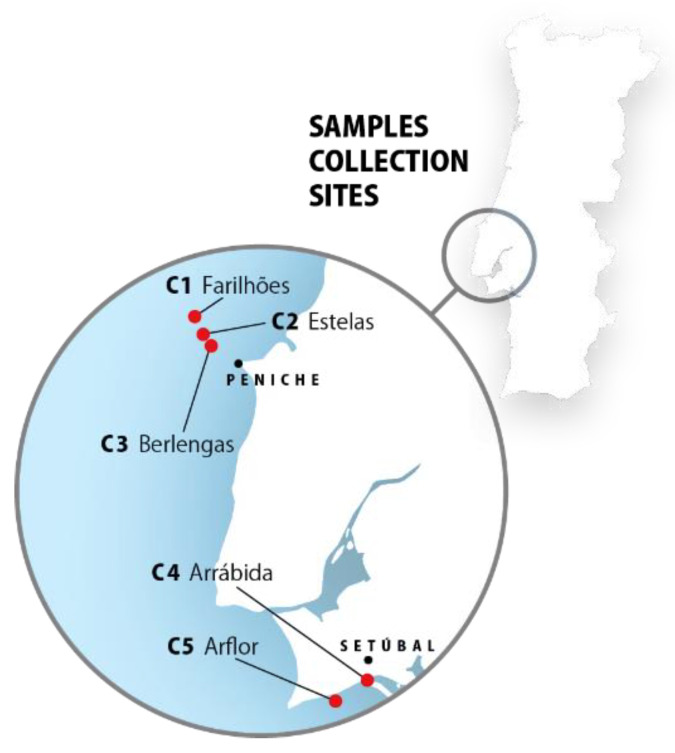
*Cliona celata* collection sites. Samples were collected in five different sites and a distinct code name was given to samples originated from each location.

The lyophilized samples (C1, C2, C3, C4, and C5) of the marine sponge *Cliona celata* (Grant, 1826) were previously obtained by MARE-FCUL team, within the scope of project LUSOEXTRAT. Samples were collected from five different sites alongside the Portuguese coast, in Arrábida and Arflor, Setúbal, and Berlengas and Farilhões islands, Peniche (Portugal) (Figure 6).

### 4.3. Marine Sponge Extraction

Each lyophilized sample (1 g) was extracted with a mixture (200 mL) of methanol (MeOH):dichloromethane (CH_2_Cl_2_) (*v*/*v*) (1:1) followed by an ultrasound-assisted extraction for 20 min, and 20 min of continuous stirring. The resulting solution was filtered with qualitative filter paper No.1 (VWR International, Alfragide, Portugal). The extraction process was repeated twice with the leftover biomass. Solution samples were concentrated until dryness, under vacuum at low temperature (40 °C), in a rotary evaporator (IKA HB10, VWR International, Alfragide, Portugal) and in a speed-vacuum equipment (Eppendorf Concentrator Plus, Leicestershire, UK). The obtained crude extracts (C1, C2, C3, C4, and C5) were used in the preliminary in vitro biological assays.

### 4.4. Sponge Extracts Fractionation

Extract C4 was selected for fractionation through a preparative column chromatography due to its high anti-inflammatory capacity. The initial mass of the extract was 3.57 g. Extract C4 and silica gel 60–200 μm (VWR, Fontenay-sous-Bois, France) were added to a MeOH: CH_2_Cl_2_ (*v*/*v*) (1:1) solution, concentrated until dryness, and fractionated using a glass column packed with silica gel 60. The elution system consisted of a *n*-hexane: ethyl acetate (NH:EA) gradient step until 100% ethyl acetate was reached, followed by a MeOH:CH_2_Cl_2_ (*v*/*v*) mixture (1:1) and 100% MeOH. The fractionation of C4 afforded a total of 16 fractions, according to the employed mobile phase (F1—100% NH, F2—95:5 NH:EA, F3—90:10 NH:EA, F4—85:15 NH:EA, F5—82.5:17.5 NH:EA, F6—80:20 NH:EA, F7—77.5:22.5 NH:EA, F8.1 and F8.2 75:25 NH:EA, F9—70:30 NH:EA, F10—60:40 NH:EA, F11—50:50 NH:EA, F12—100% EA, F13 50:50 MeOH:CH_2_Cl_2_, F14.1 and F14.2—100% MeOH).

### 4.5. Evaluation of Biological Activities of Cliona celata Crude Extracts and Fractions

For in vitro bioassays, crude extracts and fractions were dissolved in dimethyl sulfoxide (DMSO) at a concentration of 20 mg·mL^−1^. The controls were always treated with the highest tested concentration of DMSO as vehicle.

#### 4.5.1. Antioxidant Activity

The antioxidant activity of *C. celata* crude extracts was evaluated using several in vitro chemical assays, including DPPH radical scavenging activity, FRAP, and ORAC. BHT was used as an antioxidant standard.

##### 2,2-Diphenyl-1-Picrylhydrazyl (DPPH) Radical Scavenging Activity

The DPPH radical scavenging activity was performed according to Brand-Williams and co-workers [46], adapted to microplate with slight modifications [47]. Crude extracts were tested at a final concentration of 200 µg·mL^−1^. Briefly, samples (2 μL) were mixed with DPPH reagent (198 μL) (0.1 mM in ethanol) and incubated in the dark for 30 min, at room temperature. The absorbance was then measured at 517 nm (Epoch 2 microplate spectrophotometer, BioTek, Winooski, VT, USA). Results were expressed as DPPH percentage of control (DPPH % of control).

##### Ferric Reducing Antioxidant Power (FRAP)

The FRAP method was performed according to Benzie and Strain [48], adapted to microplate with slight adjustments [49]. Briefly, FRAP reagent (0.3 M acetate buffer (pH = 3.6), 10 mM 2,4,6-Tris(2-pyridyl)-*s*-triazine (TPTZ) in 40 mM HCl and 20 mM FeCl_3_ at a ratio of 10:1:1) was prepared and incubated at 37 °C for approximately 15 min. Afterward, a standard curve of FeSO_4_ (0–10 μM) was prepared. Crude extracts and standard curve concentrations (2 μL) were mixed with FRAP reagent (198 μL) and incubated in the dark for 30 min at 37 °C. Blanks for each sample and standard curve concentration were also prepared by replacing the FRAP reagent with acetate buffer. The absorbance was then measured at 593 nm. Results were expressed as micromolar of FeSO_4_ equivalents per gram of sample (µM of FeSO_4_ eq·g^−1^ of sample).

##### Oxygen Radical Absorbance Capacity (ORAC)

The ORAC assay was performed as described by Dávalos and co-workers [50]. Crude extracts were prepared in phosphate buffer (75 mM, pH 7.4) and pre-incubated (20 μL) with fluorescein (120 µL; 70 nM) for 15 min at 37 °C. A standard Trolox curve (0–80 μM) was prepared, by adding 20 μL of the standard solution instead of samples. Then, a 2,2′-Azobis(2-amidinopropane) dihydrochloride solution (60 µL; 12 mM) was added and the fluorescence (λ_excitation_ = 458 nm; λ_emission_ = 520 nm) was recorded in a microplate reader (Synergy H1 Hybrid Reader, BioTek, Winooski, VT, USA) every min for 4 h, and automatic shaking was performed prior to each reading. The results were expressed as micromol of Trolox equivalents per gram of sample (µmol of Trolox eq·g^−1^ of sample).

#### 4.5.2. Evaluation of Biological Activities of *Cliona celata* Crude Extracts and Fractions on In Vitro Cellular Models

*C. celata* crude extracts and selected fractions were evaluated for their cytotoxic and anti-inflammatory activities. The effect of the two fractions with the highest anti-inflammatory capacity (F5 and F13) on the levels of inflammatory mediators (TNF-α, IL-6 and IL-10) on RAW 264.7 macrophages was also carried out. Lipopolysaccharides (LPS) (1 μg·mL^−1^) were used as inflammation inducers, while dexamethasone (DEX) (30 μg·mL^−1^) was used as an anti-inflammatory standard. The details of each methodology are described below.

##### Cell Culture Maintenance

Experiments were performed on an in vitro model of Abelson murine leukemia virus-induced tumor RAW 264.7 macrophages (ATCC TIB-71), acquired from the American Type Culture Collection (ATCC) biobank. RAW 264.7 macrophages were culture in Dulbecco’s Modified Eagle’s Medium/Nutrient Mixture F-12 (DMEM/F-12) supplemented with 10% (*v*/*v*) fetal bovine serum (FBS), 1% antibiotic/antimycotic commercial solution (100 IU·mL^−1^ penicillin, 100 μg·mL^−1^ streptomycin), and 1% sodium pyruvate solution (100 mM). Cells were kept in a 95% moisture and 5% CO_2_ atmosphere at 37 °C. Subculture was performed according to ATCC instructions whenever cultures reached 80–85% confluence.

##### Cytotoxic Activity in RAW 264.7 Macrophages

The cytotoxic activity of *C. celata* crude extracts and fractions was evaluated on RAW 264.7 macrophages (5 × 10^4^ cells per well), after a seeding period of 16 h in 96-well plates. Only fractions with a weight superior to 11 mg were tested for their cytotoxic effect. Cells were then treated with the crude extracts (200 μg·mL^−1^) and selected fractions (100, 160, and 200 μg·mL^−1^) for 24 h. Untreated cells were used as control, and saponin was used as a cellular death positive control, representing 100% of cell death. Following the incubation period, the cytotoxic effect of samples was estimated using the 3-[4,5-dimethylthiazol-2-yl]-2,5-diphenyltetrazolium bromide (MTT) colorimetric assay, as described by Mosmann [51]. The intracellular formazan crystals were then extracted and solubilized with DMSO (100 μL) and the absorbance was measured at 570 nm. The results were expressed as the cell viability percentage of the control of untreated cells. The maximum non-cytotoxic concentration of each fraction was selected for the quantification of NO on RAW 264.7 macrophages. Fractions that still displayed a cytotoxic behavior at the minimum tested concentration (100 μg·mL^−1^) were discarded.

##### Quantification of Nitric Oxide (NO) on RAW 267.4 Cells

The inflammatory and anti-inflammatory effects of *C. celata* crude extracts and selected fractions were evaluated through the NO production assay according to Yang and co-workers [52], with slight modifications [53], on RAW 264.7 macrophages (5 × 10^4^ cells per well), after a seeding period of 16 h in 96-well plates. Cells were pre-incubated for 1 h with samples at their respective maximum non-cytotoxic concentration. Then, LPS (10 μL; 1 μg·mL^−1^) was added and the cells were incubated for 24 h to assess the anti-inflammatory effect of samples in LPS-induced RAW 264.7 macrophages. Untreated cells were used as control. Simultaneously, a situation where ultrapure water (10 μL) was added to samples instead of LPS was evaluated, to assess the ability of samples to increase NO levels in the absence of induction of inflammation on RAW 264.7 cells. A concentration-dependent analysis (0–200 μg·mL^−1^) was conducted on the extract with the highest anti-inflammatory activity.

Following the incubation period, Griess reagent was freshly prepared 30 min before use by mixing 0.1% naphthylethylene diamine dihydrochloride with 1% sulphanilamide in 2.5% phosphoric acid. Culture medium (100 μL) from each well was transferred to a new plate, and Griess reagent (100 μL) was added. The plate was then incubated in the dark at room temperature for 30 min, and the absorbance was measured at 546 nm. The percentage of nitric oxide production was calculated using the following formula:

(1) $$\mathrm{Nitric}\;\mathrm{Oxide}\;\mathrm{Production}\;(\%\;\mathrm{of}\;\mathrm{control})=\frac{Abs_{TEST}}{Abs_{CONTROL}}\times 100$$

where $Abs_{TEST}$ = absorbance values in the presence of samples in an inflammatory condition and $Abs_{CONTROL}$ = absorbance values of the control situation, where inflammation was not induced.

Results were expressed as nitric oxide production percentage of control [Nitric Oxide Production (% of Control)].

##### Quantification of the Levels of TNF-α, IL-6, and IL-10 on LPS-Induced RAW 264.7 Macrophages

The two fractions with the highest anti-inflammatory activity (F5 and F13) were selected to assess their effects on the levels of TNF-α, IL-6, and IL-10 on LPS-induced RAW 264.7 macrophages, through an enzyme-linked immunosorbent assay (ELISA). RAW 264.7 macrophages were cultured in 24-well plates (5 × 10^5^ cells per well), for a seeding period of 16 h. Cells were pre-incubated for 1 h with samples (990 μL) at their respective maximum non-cytotoxic concentration. Then, LPS (10 μL; 1 μg·mL^−1^) was added and the cells were incubated for 18 h for the quantification of the cytokine release of TNF-α, IL-6, and IL-10, respectively. Following each incubation period, 500 μL of cell supernatant was collected and stored at −80 °C (Thermo Electron Corporation, Waltham, MA, USA) until the quantification of the cytokines release was conducted.

Mouse TNF-α Uncoated ELISA, mouse IL-6 Uncoated ELISA, and mouse IL-10 Uncoated ELISA (887324-22, 88-7064-22 and 88-7105-22, respectively, Thermo Fisher Scientific, Vilnius, Lithuania) kits were purchased for the immunoassays. ELISA was performed according to the manufacturers’ instructions with slight adjustments. Values were read at 570 and 450 nm to allow wavelength subtraction. Results were expressed as TNF-α, IL-6, and IL-10 concentrations (pg·mL^−1^).

### 4.6. Chemical Characterization by GC-MS Analysis

The most bioactive samples, F5 and F13, were analyzed by GC-MS using a Shimadzu QP2010-Plus GC/MS system according to the previously reported procedure [54]. A tentative identification of the major volatilized compounds of F5 and F13 fractions was performed by matching the mass fragmentation patterns with those in the GC-MS mass spectral databases (Wiley 229 and NIST-National Institute of Standards and Technology libraries).

### 4.7. Data and Statistical Analysis

One-way analysis of variance (ANOVA) with Dunnett’s multiple comparison of group means to determine significant differences with the control treatment was applied. When required, Tukey’s test was performed to assess the statistical differences between the mean of samples in study. All data were checked for normality and homoscedasticity. The results are presented as mean ± standard error of the mean (SEM). Statistical differences were considered at the significance level of 0.05 (*p* < 0.05). At least three independent experiments were carried out in triplicate. Calculations were performed using IBM SPSS Statistics 24 (IBM Corporation, Armonk, NY, USA) and GraphPad v5.1 (GraphPad Software, La Jolla, CA, USA) software.

## Figures and Tables

**Table 1:** Antioxidant capacity of *Cliona celata* crude extracts (C1–C5).

Extract	DPPH ^1^	FRAP ^2^	ORAC ^3^
C1	96.56 ± 0.85	24.32 ± 2.24	173.63 ± 7.07
C2	95.56 ± 1.63	31.93 ± 5.45	120.22 ± 5.24
C3	94.35 ± 1.02	35.41 ± 3.62	248.91 ± 6.74
C4	89.98 ± 1.18	77.63 ± 4.10	239.37 ± 7.50
C5	98.04 ± 0.71	38.54 ± 3.38	171.67 ± 6.17
BHT	39.10 ± 2.97	2821.50 ± 63.04	136.38 ± 9.09

^1^ Radical scavenging activity (% of DPPH); ^2^ FeSO_4_ equivalents per g of extract (μM FeSO_4_ eq·g^−1^); ^3^ Trolox equivalents per g of extract (µmol T eq·g^−1^).

**Table 2:** Extraction yields (%) and effect on cell viability of *Cliona celata* fractions on RAW 264.7 macrophages.

Fraction	Yield (%)	Cells Viability (% of Control)		
		200 μg·mL^−1^	160 μg·mL^−1^	100 μg·mL^−1^
F1	0.08	-	-	-
F2	0.05	-	-	-
F3	0.11	-	-	-
F4	0.63	-	-	-
F5	7.29	64.41 ± 7.23 *	118.35 ± 2.83	101.00 ± 7.16
F6	4.90	7.31 ± 2.89 *	19.13 ± 2.84 *	46.83 ± 2.09 *
F7	2.32	0.00 ± 0.00 *	15.18 ± 3.96 *	36.76 ± 3.46 *
F8.1	1.47	0.00 ± 0.00 *	4.71 ± 1.36 *	15.69 ± 1.88 *
F8.2	5.60	0.00 ± 0.00 *	22.99 ± 3.81 *	29.84 ± 3.76 *
F9	3.58	0.08 ± 0.09 *	8.80 ± 1.72 *	50.19 ± 11.71 *
F10	0.87	-	-	-
F11	7.75	24.33 ± 7.65 *	32.97 ± 5.84 *	63.64 ± 3.79 *
F12	7.95	4.81 ± 2.07 *	18.08 ± 4.06 *	50.59 ± 3.51 *
F13	6.21	8.45 ± 1.71 *	68.62 ± 2.98 *	82.61 ± 4.46
F14.1	30.25	82.50 ± 4.03 *	79.91 ± 4.09 *	68.39 ± 8.09 *
F14.2	14.25	82.30 ± 3.13 *	71.40 ± 1.83 *	61.43 ± 3.95 *

* Significant differences (one-way ANOVA, Dunnett’s test; *p* < 0.05) when compared to the vehicle.

## Supplementary Materials

The following are available online at https://www.mdpi.com/article/10.3390/md19110632/s1, Figure S1: GC-MS analysis of fractions F5 and F13.

## References

[1] Du C., Bhatia M., Tang S.C.W., Zhang M., Steiner T. (2015). Mediators of Inflammation: Inflammation in Cancer, Chronic Diseases, and Wound Healing. Mediators Inflamm..

[2] Chen L., Deng H., Cui H., Fang J., Zuo Z., Deng J., Li Y., Wang X., Zhao L. (2018). Inflammatory responses and inflammation-associated diseases in organs. Oncotarget.

[3] Netea M.G., Balkwill F., Chonchol M., Cominelli F., Donath M.Y., Giamarellos-Bourboulis E.J., Golenbock D., Gresnigt M.S., Heneka M.T., Hoffman H.M. (2017). A guiding map for inflammation. Nat. Immunol..

[4] Duque G.A., Descoteaux A. (2014). Macrophage cytokines: Involvement in immunity and infectious diseases. Front. Immunol..

[5] Turner M.D., Nedjai B., Hurst T., Pennington D.J. (2014). Cytokines and chemokines: At the crossroads of cell signalling and inflammatory disease. Biochim. Biophys. Acta.

[6] Rea I.M., Gibson D.S., McGilligan V., McNerlan S.E., Denis Alexander H., Ross O.A. (2018). Age and age-related diseases: Role of inflammation triggers and cytokines. Front. Immunol..

[7] Coleman J.W. (2001). Nitric oxide in immunity and inflammation. Int. Immunopharmacol..

[8] Sharma J.N., Al-Omran A., Parvathy S.S. (2007). Role of nitric oxide in inflammatory diseases. Inflammopharmacology.

[9] Hunter P. (2012). The inflammation theory of disease. EMBO Rep..

[10] Wojdasiewicz P., Poniatowski Ł.A., Szukiewicz D. (2014). The role of inflammatory and anti-inflammatory cytokines in the pathogenesis of osteoarthritis. Mediat. Inflamm..

[11] Pizzino G., Irrera N., Cucinotta M., Pallio G., Mannino F., Arcoraci V., Squadrito F., Altavilla D., Bitto A. (2017). Oxidative Stress: Harms and Benefits for Human Health. Oxid. Med. Cell. Longev..

[12] Liguori I., Russo G., Curcio F., Bulli G., Aran L., Della-Morte D., Gargiulo G., Testa G., Cacciatore F., Bonaduce D. (2018). Oxidative stress, aging, and diseases. Clin. Interv. Aging.

[13] Hwang J.H., Ma J.N., Park J.H., Jung H.W., Park Y.K. (2018). Anti-inflammatory and antioxidant effects of MOK, a polyherbal extract, on lipopolysaccharide-stimulated RAW 264.7 macrophages. Int. J. Mol. Med..

[14] Siti H.N., Kamisah Y., Kamsiah J. (2015). The role of oxidative stress, antioxidants and vascular inflammation in cardiovascular disease (a review). Vasc. Pharmacol..

[15] El-Demerdash F.M., Tousson E.M., Kurzepa J., Habib S.L. (2018). Xenobiotics, oxidative stress, and antioxidants. Oxidative Med. Cell. Longev..

[16] Stonik V.A. (2009). Marine Natural Products: A Way to New Drugs. Acta Naturae.

[17] Mehbub M.F., Lei J., Franco C., Zhang W. (2014). Marine sponge derived natural products between 2001 and 2010: Trends and opportunities for discovery of bioactives. Mar. Drugs.

[18] Anjum K., Abbas S.Q., Shah S.A.A., Akhter N., Batool S., Hassan S.S.U. (2016). Marine sponges as a drug treasure. Biomol. Ther..

[19] Kim S.K. (2015). Springer Handbook of Marine Biotechnology.

[20] Yang J.H., Suh S.J., Lu Y., Li X., Lee Y.K., Chang Y.C., Na M.K., Choi J.H., Kim C.H., Son J.K. (2010). Anti-inflammatory activity of ethylacetate fraction of *Cliona celata*. Immunopharmacol. Immunotoxicol..

[21] Keyzers R.A., Daoust J., Davies-Coleman M.T., Van Soest R., Balgi R., Donohue E., Roberge M., Andersen R.J. (2008). Autophagy-modulating aminosteroids isolated from the sponge *Cliona celata*. Org. Lett..

[22] Gad S.C. (2005). Drug Discovery Handbook.

[23] Hong J. (2011). Natural product diversity and its role in chemical biology and drug discovery. Curr. Opin. Chem. Biol..

[24] Seradj H., Moein M., Eskandari M., Maaref F. (2012). Antioxidant Activity of Six Marine Sponges Collected from the Persian Gulf. Iran. J. Pharm. Sci..

[25] Shaaban M., Abd-Alla H.I., Hassan A.Z., Aly H.F., Ghani M.A. (2012). Chemical characterization, antioxidant and inhibitory effects of some marine sponges against carbohydrate metabolizing enzymes. Org. Med. Chem. Lett..

[26] Bary K., Elamraoui B., Bamhaoud T. (2016). Chemical characterization of Cliona viridis: Sponge of Atlantic Moroccan Coast. Int. J. Innov. Sci. Res..

[27] Yuan G., Wahlqvist M.L., He G., Yang M., Li D. (2006). Natural products and anti-inflammatory activity. Asia Pac. J. Clin. Nutr..

[28] Fung S.Y., Sofiyev V., Schneiderman J., Hirschfeld A.F., Victor R.E., Woods K., Piotrowski J.S., Deshpande R., Li S.C., De Voogd N.J. (2013). Unbiased screening of marine sponge extracts for antiinflammatory agents combined with chemical genomics identifies girolline as an inhibitor of protein synthesis. ACS Chem. Biol..

[29] El-Demerdash A., Atanasov A.G., Horbanczuk O.K., Tammam M.A., Abdel-Mogib M., Hooper J.N.A., Sekeroglu N., Al-Mourabit A., Kijjoa A. (2019). Chemical diversity and biological activities of marine sponges of the genus *Suberea*: A systematic review. Mar. Drugs.

[30] Koelman L., Pivovarova-Ramich O., Pfeiffer A.F.H., Grune T., Aleksandrova K. (2019). Cytokines for evaluation of chronic inflammatory status in ageing research: Reliability and phenotypic characterisation. Immun. Ageing.

[31] Kinne R.W., Stuhlmüller B., Burmester G.R. (2009). Macrophages. Rheumatoid Arthritis.

[32] Ravussin E., Smith S.R. (2015). Role of the Adipocyte in Metabolism and Endocrine Function. Endocrinology: Adult and Pediatric.

[33] Gabay C. (2006). Interleukin-6 and chronic inflammation. Arthritis Res. Ther..

[34] Tanaka T., Narazaki M., Kishimoto T. (2014). Il-6 in inflammation, Immunity, and disease. Cold Spring Harb. Perspect. Biol..

[35] Barnes P.J. (2001). Cytokine modulators as novel therapies for airway disease. Eur. Respir. J..

[36] Iyer S.S., Cheng G. (2012). Role of interleukin 10 transcriptional regulation in inflammation and autoimmune disease. Crit. Rev. Immunol..

[37] Di X., Oskarsson J.T., Omarsdottir S., Freysdottir J., Hardardottir I. (2017). Lipophilic fractions from the marine sponge *Halichondria sitiens* decrease secretion of pro-inflammatory cytokines by dendritic cells and decrease their ability to induce a Th1 type response by allogeneic CD4+ T cells. Pharm. Biol..

[38] Costantini S., Romano G., Rusolo F., Capone F., Guerriero E., Colonna G., Ianora A., Ciliberto G., Costantini M. (2015). Anti-Inflammatory Effects of a Methanol Extract from the Marine Sponge *Geodia cydonium* on the Human Breast Cancer MCF-7 Cell Line. Mediat. Inflamm..

[39] Prabakaran R., Kumar T.S., Rao M.V. (2014). GC-MS Analysis and In vitro Cytotoxicity Studies of Root Bark Exudates of *Hardwickia binata* Roxb. Am. J. Phytomed. Clin. Ther..

[40] Stonard R.J., Andersen R.J. (1980). Celenamides A and B, Linear Peptide Alkaloids from the Sponge *Cliona celata*. J. Org. Chem..

[41] Stonard R.J., Andersen R.J. (1980). Linear peptide alkaloids from the sponge *Cliona celata* (Grant). Celenamides C and D. Can. J. Chem..

[42] Lenis L.A., Nuñez L., Jiménez C., Riguera R. (1996). Isonitenin and acetylhomoagmatine new metabolites from the sponges *Spongia officinalis* and *Cliona celata* collected at the galician coast (NW Spain). Nat. Prod. Lett..

[43] Castellanos L., Duque C., Rodríguez J., Jiménez C. (2006). Synthesis of acetylhomoagmatine. Mar. Drugs.

[44] Siodłak D. (2015). α,β-Dehydroamino acids in naturally occurring peptides. Amino Acids.

[45] Ruocco N., Costantini S., Costantini M. (2016). Blue-print autophagy: Potential for cancer treatment. Mar. Drugs.

[46] Brand-Williams W., Cuvelier M.E., Berset C. (1995). Use of a free radical method to evaluate antioxidant activity. LWT Food Sci. Technol..

[47] Pinteus S., Silva J., Alves C., Horta A., Fino N., Rodrigues A.I., Mendes S., Pedrosa R. (2017). Cytoprotective effect of seaweeds with high antioxidant activity from the Peniche coast (Portugal). Food Chem..

[48] Benzie I.F.F., Strain J.J. (1967). The ferric reducing ability of plasma (FRAP) as a measure of “antioxidant power”: The FRAP assay. J. Lab. Clin. Med..

[49] Silva J., Alves C., Freitas R., Martins A., Pinteus S., Ribeiro J., Gaspar H., Alfonso A., Pedrosa R. (2019). Antioxidant and neuroprotective potential of the brown seaweed *Bifurcaria bifurcata* in an in vitro Parkinson’s disease model. Mar. Drugs.

[50] Dávalos A., Gómez-Cordovés C., Bartolomé B. (2003). Extending Applicability of the Oxygen Radical Absorbance Capacity (ORAC-Fluorescein) Assay. Agric. Food Chem..

[51] Mosmann T. (1983). Rapid colorimetric assay for cellular growth and survival: Application to proliferation and cytotoxicity assays. J. Immunol. Methods.

[52] Yang E.J., Yim E.Y., Song G., Kim G.O., Hyun C.G. (2009). Inhibition of nitric oxide production in lipopolysaccharide-activated RAW 264.7 macrophages by Jeju plant extracts. Interdiscip. Toxicol..

[53] Freitas R., Martins A., Silva J., Alves C., Pinteus S., Alves J., Teodoro F., Ribeiro H.M., Gonçalves L., Petrovski Ž. (2020). Highlighting the biological potential of the brown seaweed *Fucus spiralis* for skin applications. Antioxidants.

[54] Silva J., Martins A., Alves C., Pinteus S., Gaspar H., Alfonso A., Pedrosa R. (2020). Natural Approaches for Neurological Disorders-The Neuroprotective Potential of *Codium tomentosum*. Molecules.

