# Supplementary material for: Unravelling the Anti-Inflammatory and Antioxidant Potential of the Marine Sponge Cliona celata from the Portuguese Coastline
Source: Mar Drugs. 2021 Nov 12;19(11):632. doi: 10.3390/md19110632 (PMC8625174; doi:10.3390/md19110632)
Supplement: Supplementary file 1 [file marinedrugs-19-00632-s001.zip › marinedrugs-1431384-supplementary.pdf]

Supplementary Information (SI1)

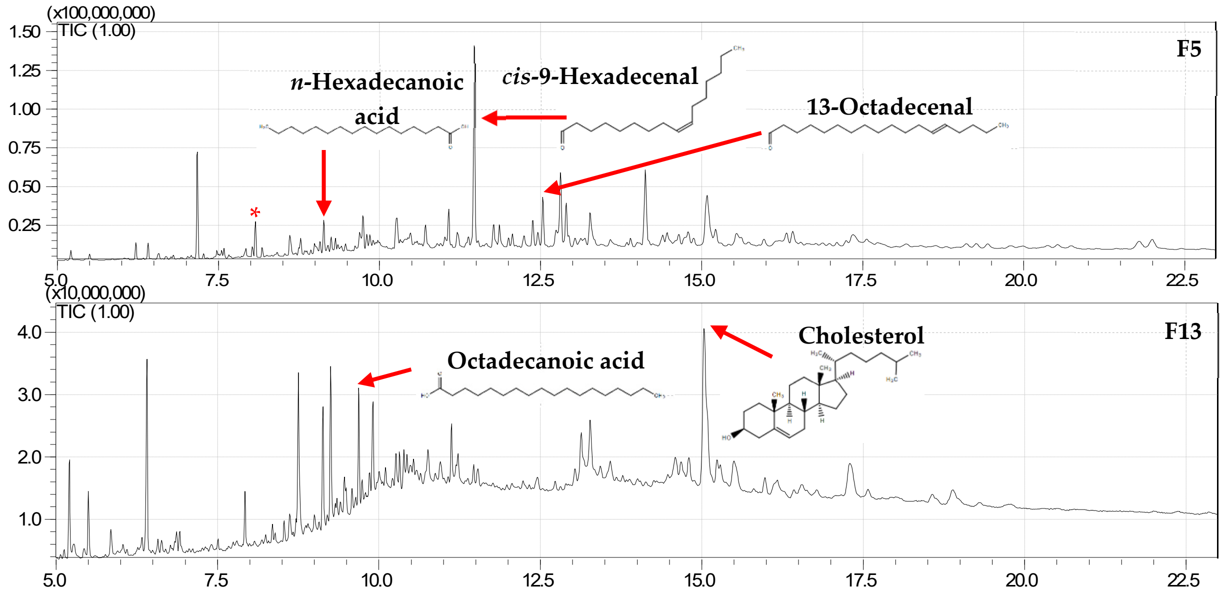

Figure S1. GC-MS analysis of fractions F5 and F13. Compounds were identified by matching the mass fragmentation patterns with those present in the GC-MS mass spectral databases (Wiley 229 and NIST-National Institute of Standards and Technology libraries). *Contaminant of the GC analysis (cyclooctasiloxane).
